# Quantum DFT methods to explore the interaction of 1-Adamantylamine with pristine, and P, As, Al, and Ga doped BN nanotubes

**DOI:** 10.1038/s41598-022-24200-x

**Published:** 2022-11-19

**Authors:** Ebrahim Nemati‐Kande, Amir Pourasadi, Fatemeh Aghababaei, Samaneh Baranipour, Ata Mehdizadeh, Jaber Jahanbin Sardroodi

**Affiliations:** 1 Department of Physical Chemistry, Faculty of Chemistry, Urmia University, Urmia, Iran; 2 Department of Chemistry, Faculty of Basic Sciences, Azarbaijan Shahid Madani University, 35th km of Tabriz-Maragheh Road, Tabriz, Iran; 3 Molecular Simulation Laboratory, Azarbaijan Shahid Madani University, Tabriz, Iran; 4 Molecular Science and Engineering Research Group (MSERG), Azarbaijan Shahid Madani University, Tabriz, Iran

**Keywords:** Materials chemistry, Physical chemistry, Theoretical chemistry

## Abstract

Nanostructures, nowadays, found growing applications in different scientific and industrial areas. Nano-coins, nanosheets, and nanotubes are used in medical applications as sensors or drug delivery substances. The aim of this study is to explore the adsorption of 1-Adamantylamine drug on the pristine armchair boron nitride nanotubes (BNNTs) with BNNT(5,5), BNNT(6,6), and BNNT(7,7) chirality along with the P, As, Al and Ga-doped BNNTs, using the quantum mechanical density functional methods. Considering the fact that dispersion effects are important in the case of weak Van der Waals interactions, computations have been done using B3LYP hybrid functional with the implementation of the D3(BJ) empirical dispersion correction methods. Quantum theory of atoms in molecules, natural bonding orbitals, and Kohn–Sham orbitals were used to investigate the nature and type of the adsorption process. The results showed that, while the adsorption of 1-Adamantylamine on the outer surface of pristine BNNT is physical in nature, doping can improve the ability of detracted BN to adsorb the drug through chemical bonds. Also, it was found that, by increasing the radius of the BNNT the adsorption energy was decreased. In conclusion, results of the present work suggest that, Ga doped nanotube, due the chemisorption, is not an ideal nanotube in drug delivery of 1-Adamantylamine drug, whereas, the other studied cases physiosorbed the drug, and may not have serious problem in release of the 1-Adamantylamine drug.

## Introduction

Carbon nanotubes are promising candidates as drug delivery carriers for anti-cancer drugs^1,2^ to pass through the cell walls^3^, however, their toxicity may cause some systematic problems^4^. Therefore, searching for alternative materials with similar geometric properties and greater chemical and physical resistance may be an alternative solution to such problems. Boron nitride nanotubes (BNNTs), were first synthesized experimentally in 1995 by Chopra and coworkers^5^, and may be an excellent alternative to CNTs, because of similar geometrical structure, similar mechanical properties^6,7^, and high thermal and chemical stability^8^. BNNTs are non-toxic^9,10^ biocompatible^11^, hydrophobic^12^ chemically neutral, and inactive to DNA^13^, and therefore are optimistic drug delivery carriers.

Such interesting features have affected the attention of some research to study the use of BNNTS in drug delivery and immobilization of drugs and biological substances. In this respect, Mukhopadhyay and coworkers^14^ showed that BNNTs are more sensitive than CNTs toward amino acid polarity. The effect of increasing the nanotube size was studied by Saikia et al.^15^. Their results showed that, by increasing the radius of the nanotube the adsorption energy of Isoniazid drug on BNNT was increased. Peyghan et al.^16^ also reported that BNNT can effectively adsorb Imidazole drug and is a good drug carrier, especially in the presence of polar solvents. Yang^17^ suggested that BNNT may be used as a nanoscale smooth channel to transfer a broad range of small biological molecules. Anota et al.^18^ studied the adsorption of metformin drug on the single wall armchair BNNT, and found that, the chemical reactivity of complex was increased by 0.42 eV compared to the individual substances.

Soltani et al.^19^ reported a systematic theoretical investigation of the interaction of the Fluorouracil (5-FU) molecule with single-walled zigzag BNNTs doped with Gallium, Germanium, and Aluminum metallic atoms. The interaction of noble gases and CH_3_X (X = F, Cl and Br) gases^20^ with pristine and decorated nanotubes was has studied by our group. Adamantane with the chemical formula of C_10_H_16_ is an organic polyhedral compound and is the structural backbone of some chemical compounds with potential applications in clinical practices^21^. 1-adamantylamine is an adamantane derivative in which an amino group was replaced by one of four methyl groups of adamantane. 1-Adamantylamine was first used to treat the flu virus due to its antiviral properties, it is now mainly used in the treatment of Parkinson^22^, and some types of virus^23^.

Quantum mechanical density functional theory (DFT) methods have found a wide range of applications, especially in the field of predicting the properties of nanomaterials^24–27^, separation and purification of chemicals^28–30^, prediction of the mechanism of reactions^31^, and so on. Recently, some important methods such as functionalization/doping and size-dependent were used to enhance or control the material properties. Among them, doping process has been the subject of intensive research to make suitable material properties for use in a wide range of applications^32–36^.

In this work, DFT methods using PBE^37^ and B3LYP–D3(BJ)^38,39^ functionals were implemented to explore the interaction of 1-Adamantanamine with BNNT. Also, the BNN nanotube was doped with Al and Ga metallic elements, and P, and As non-metallic compounds to activate the surface of BNNT to 1-adamantylamine. Buder’s quantum theory of atom in molecules (QTAIM) ^40^, natural bond orbital, NBO ^41^, Density of state (DOS) spectra, and Kohn–sham molecular orbitals were also used to more deep analysis of the adsorption properties of 1-adamantylamine on BNNT.

## Computational methods

Boron Nitride nanotubes (BNNT) with (5,5), (6,6), and (7,7) chirality with B_40_N_40_, B_48_N_48,_ B_56_N_56_ chemical formulas were used as an initial unit-cell. The PBE-PBE^37^ and B3LYP^38,39^ hybrid exchange–correlation functionals were implemented. Both functional are obtained from the linear-combination of Hartree–Fock (HF) exchange functional with other functionals. Indeed, in the PBE-PBE functional the contribution of HF exchange energy is 33.3%, and the other remaining exchange energy is from the PBE functional^37^, where, the correlation energy is totally from PBE functional. However, in B3LYP hybrid functional the contribution of HF exchange energy is about 8%.

Then, the PBE functional of Pedrew et al.^37^ with the implementation of periodic boundary condition (PBC) was used to optimize the unit-cells. The PBC condition was implemented in one direction along the tube axis, and the 6-311G* basis set was also used.

After optimization of the pristine BNNT(5,5) metallic Al or Ga atom was substituted at one of the central Boron atoms to obtain the doped BN(Al) or BN(Ga) nanotubes. In a similar manner a central N atom of the pristine BNNT was replaced by non-metallic P, or As atom as a dopant to produce decorated BN(P) or BN(As) nanotube, respectively. The main reason for choosing these elements is that, they are in the same chemical group as the elements of the BNNT(5,5), and as a result of having the same valence electrons, doped BNNTs are isoelectronic to the primary BNNT(5,5) nanotube. The decorated BN(Al), BN(Ga), BN(P), and BN(As) nanotubes also were reoptimized using the similar DFT-PBC method. Because, the studied nanotubes are closed-shell, only odd spin-multiplicity states are possible. Therefore, to consider the possible spin-multiplicities, the singlet and triplet spin-multiplicity states were considered, and the energy of the nanotubes were calculated at PBE-PBE/6-311G* level. The results were summarized in Table 1. It is obvious that, in all cases the singlet state is about 3.7–4.5 eV more stable than the triplet state, and therefore, the other calculations were done using the singlet spin-multiplicity. 1-adamantylamine (ADA) has also been optimized using this method, and the optimized structure was used to produce the ADA/nanotube complexes at different positions.

**Table 1:** Energies of the singlet (E_S_) and triplet (E_T_) spin-multiplicity states for the studied nanotubes, calculated at PBE-PBE/6-311G* level of theory, and with applying PBC.

Nanotube	E_S_ (a.u.)	E_T_ (a.u.)	E_S_-E_T_ (eV)
BNNT(5,5)	− 3184.982228	− 3184.815362	− 4.54
BNNT(Al)	− 3402.401335	− 3402.253616	− 4.02
BNNT(Ga)	− 5082.687487	− 5082.551242	− 3.71
BNNT(P)	− 3471.367688	− 3471.220900	− 3.99
BNNT(As)	− 5363.595038	− 5363.451449	− 3.91

The cohesion energy (*E*_*coh*_) of the studied nanotubes was also calculated by expanding the optimized nanotube 4 times in the applied-direction of the PBC box, elimination the PBC, and saturating the terminal atoms with H atoms. Therefore, the expanded BNNT(5,5), BNNT(6,6), and BNNT(7,7) with the chemical formula of B_160_N_160_H_20_, B_192_N_192_H_24_, and B_224_N_224_H_28_ was obtained. *E*_*coh*_ was calculated using the following relation:

1 $$E_{coh} = (E_{NT} - n_{N} \mu_{N} - n_{B} \mu_{B} - n_{H} \mu_{H} - n_{D} \mu_{D} )/(n_{N} + n_{B} + n_{H} + n_{D} )$$

in which, *E*_*NT*_ is the energy of the optimized nanotube, *n*_*N*_, *n*_*B*_*, n*_*H*_*,* and *n*_*D*_ are the number of N, B, H, and dopped atoms (i.e., Al, Ga, P, or As), and *μ* is the chemical potential of the relevant atom, i.e., the energy of the free atom in vacuum.

Furthermore, given the symmetric constraints, there are only four possible adsorption cites on pristine BNNT; i.e., T1: the center of hexagonal ring, T2: on top of Boron atom, T3: on top of Nitrogen atom, and T4: the middle of B–N bond. For ADA, however, there is two possible different cites from a chemical point of view; i.e., the methine position, and the amino group. The doped nanotubes also similar to pristine BNNT other than the doped atom. Therefore, all possible configurations for ADA/nanotube complexes were produced by placing ADA from methine or amino groups on T1 to T4 positions of pristine BNNT with 2 Å vertical distance between ADA and BNNT, and the complex was reoptimized at PBE-PBE/6-311G^*^ level of theory by applying 1-dimentional PBC. Obtained results showed that, after optimization of the complexes composed from methine side of ADA, the ADA molecule rotated and absorbed from amino group on BNNT, and the final structure was exactly on top of the T1-T4 states. Therefore, in the case of doped nanotubes, ADA from only amino group was placed on top of the doped atom with vertical distance of 2 Å and the resulting structures were reoptimized. All optimizations and NBO calculation were done using Gaussian 16^42^ suit of programs. QTATM analysis and DOS spectra was calculated using Multiwfn program^43^. To do NBO and QTAIM analysis the unit calls were three times expended in the applied PBC direction, the PBC was eliminated and the boundary atoms were terminated with hydrogen atoms too keep the system closed shell.

Although PBE-PBE functional is slightly fast in optimization of the structures, and the optimized structures using this function were shown^20,44^ to be in consistence with the experimental results; however, its applicability in the calculation and analysis of energetic parameters is questionable, mainly due to the elimination of the long-range dispersion effects, which may be large specially in the case of weak Van der Waals (vdW) type interactions. Therefore, single point calculations on the optimized and expanded systems have also been done at the B3LYP-D3(BJ)/6-311G* level of theory to consider the long-range dispersion effects by using the third version of Grimm’s empirical dispersion correction method^39^. All other calculations have been done on the expanded systems at B3LYP-D3(BJ)/6-311G* level of theory. The adsorption Energy (*E*_*ads*_) was calculated using the following equation:

2 $$E_{ads} = E_{NT/ADA} - E_{NT} - E_{ADA}$$

In which, *E*_*NT*_ and *E*_*ADA*_ are the energies of the individual nanotube and ADA molecule, respectively, and *E*_*NT/ADA*_ is the energy of corresponding nanotube/ADA complex.

The following set of equations were also used to calculate chemical potential (*μ*), chemical hardness (*η*), and electrophilicity (*ω*) using energies of the highest occupied molecular orbital (*ε*_*H*_), and the lowest unoccupied molecular orbital (*ε*_*L*_):

3 $$\mu = \frac{1}{2}\left( {\varepsilon_{L} + \varepsilon_{H} } \right)$$

4 $$\eta = \frac{1}{2}\left( {\varepsilon_{L} - \varepsilon_{H} } \right)$$

5 $$\omega = \frac{{\mu^{2} }}{\eta }$$

## Results and discussions

### Optimized structures

**Figure 1:**
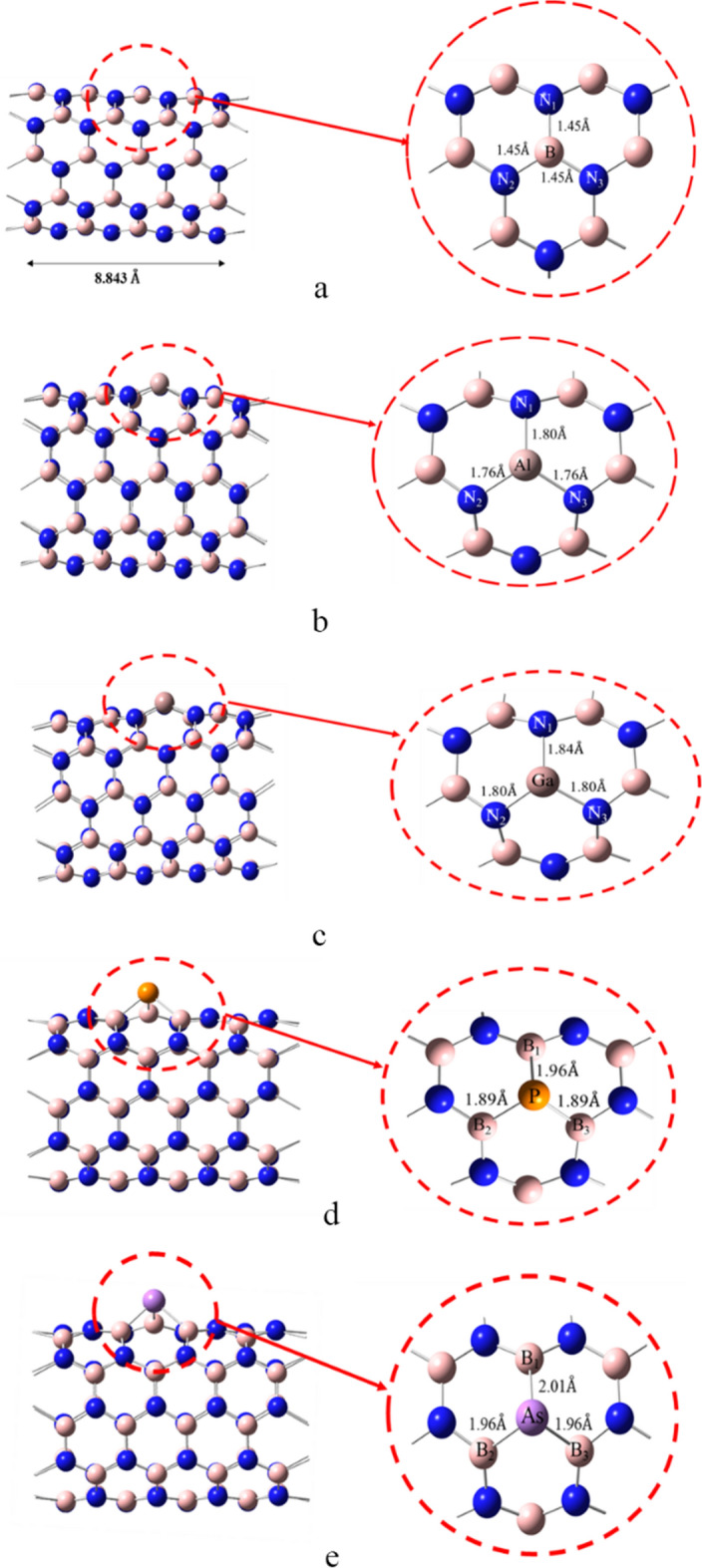
The unit-cells of (**a**) pristine BNNT, (**b**) BN(Al), (**c**) BN(Ga), (**d**) BN(P) and (**e**) BN(As) optimized at PBE-PBE/6-311G* level of theory.

**Figure 2:**
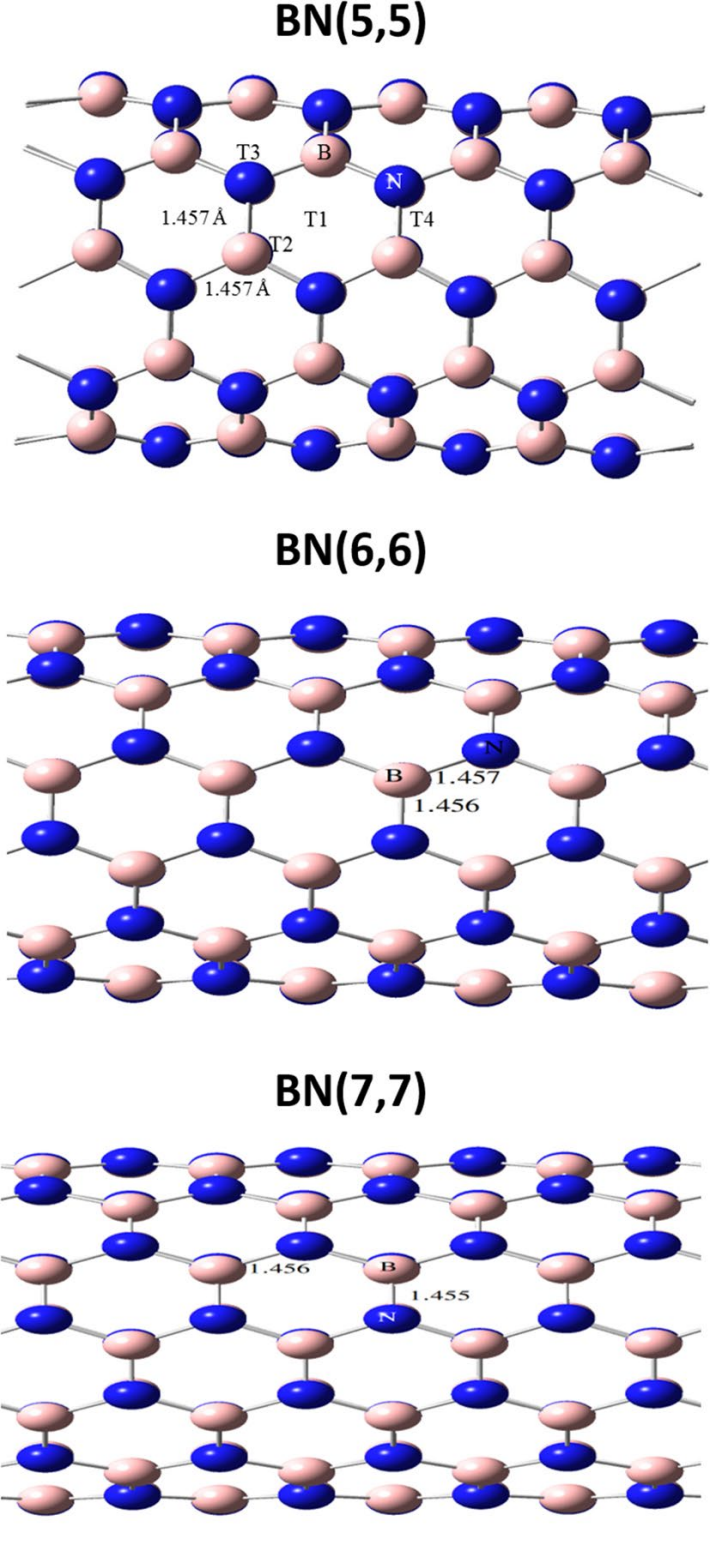
Different adsorption cites on BNNT(5,5),and comparison of the optimized pristine armchair BNNT(5,5), BNNT(6,6), and BNNT(7,7), at PBE-PBE/6-311G* level of theory, and considering 1-D periodic boundary condition.

The structure of BNNT(5,5) and doped nanotubes, which were optimized at PBE-PBE/6-311G* level of theory by applying 1-dimansional boundary conditions, were shown in Fig. 1. The B-N bond length for the optimized BNNT(5,5) was found to be 1.457 Å, which is fully in agreement with the experimental value of 1.452 Å reported in literature^45,46^. Also, the structure of the optimized pristine BNNT(5,5), BNNT(6,6), and BNNT(7,7) were compared with each other in Fig. 2. It is seen from this figure that, the bond length of the BNNT(6,6), and BNNT(7,7) are also in the range of 1.455–1.457 Å.

The cohesion energy (*E*_*coh*_) of the studied nanotubes were calculated using PBE-PBE, B3LYP, and B3LYP-D3(BJ) functionals, and both 6-31G*, and 6-311G* basis sets. The calculated *E*_*coh*_ values from different methods were reported in Table 2. All values are considerable negative values, (i.e., about − 7.0 to − 7.9 eV/atom), which confirm the stability of the constructed nanotubes. The *E*_*coh*_ at PBE-PBE/6-31G* level of theory for BNNT(5,5) was also found to be − 7.60 eV/atom, which is fully in agreement with the previous reported value of − 7.59 eV/atom obtained by HSEh1PBE/6–31g(d) method ^47^. The values obtained from PBE-PBE functionals is ~ − 0.43 eV/atom more negative than the B3LYP results, which may be due to the more contribution of the Hartree–Fock exchange correlation in PBE-PBE compared to the B3LYP functional. Form Table 2 it obvious that, the *E*_*coh*_ of the pristine BNNT are more negative for larger BNNTs, which shows that, the stability of BNNTs were increased by increasing the size of the nanotube. Also, the *E*_*coh*_ of the dopped BNNT(5,5) nanotubes is slightly (~ 0.06 eV/atom) more positive than the pristine BNNT(5,5). In other words, doping of the other atoms impose a slight instability in the BNNT, which may be related to the larger vdW radius of the doped atoms. Also, effect of different basis-sets can be inferred from this table, so that, the *E*_*coh*_ was decreased for larger basis-sets. Moreover, it is seen that, inclusion of the D3(BJ) empirical dispersion correction in B3LYP functional has increased the stability by − 0.10 eV/atom compared to the B3LYP functional.

**Table 2:** The cohesion energy (*E*_*coh*_) of the studied nanotubes in this work obtained by different theoretical methods.

Nanotube	*E*_*coh*_(eV/atom)				
	PBE-PBE/6-31G*	PBE-PBE/6-311G*	B3LYP/6-31G*	B3LYP/6-311G*	B3LYP-D3(BJ)/6-311G*
BNNT(5,5)	− 7.60	− 7.53	− 7.15	− 7.09	− 7.20
BNNT(6,6)	− 7.63	− 7.57	− 7.18	− 7.13	− 7.22
BNNT(7,7)	− 7.88	− 7.80	− 7.44	− 7.37	− 7.48
BNNT(5,5)(Al)	− 7.54	− 7.47	− 7.10	− 7.05	− 7.15
BNNT(5,5)(Ga)	− 7.54	− 7.47	− 7.10	− 7.03	− 7.14
BNNT(5,5)(P)	− 7.54	− 7.47	− 7.10	− 7.04	− 7.15
BNNT(5,5)(As)	− 7.54	− 7.46	− 7.10	− 7.03	− 7.14

The boron nitride nanotube is composed of several symmetric hexagons, that have four different adsorption positions for the adsorption of ADA onto the outer surface of the nanotube as shown in Fig. 2; i.e., adsorption position at the hexagonal center (T1); adsorption position on B atom (T2); adsorption position on N atom (T3); and the adsorption position on B-N bond (T4).

ADA molecule has also been optimized at the same level of theory, and placed on T1-T4 positions for pristine BNNT(5,5), and on top of the dopant atom in the case of doped nanotubes, as described above, and the obtained ADA/nanotube complexes reoptimized. Also, the adsorption of ADA on BNNT(6,6) and BNNT(7,7) armchair BNNTS were studied, to consider the effect of the radius and chirality of nanotubes. For BNNT(6,6), and BNNT(7,7), only the T4 position (i.e., with the most negative *E*_*ads*_) was considered. The *E*_*ads*_ values were calculated at PBE-PBE/6-311G* level of theory, and are reported in Table 3. As can be seen from this table, in all cases, other than ADA/BN(Ga) complex, *E*_*ads*_ values are in the range of physical adsorption through the weak Van der Waals (vdW) interactions. The result of this work confirms the applicability of PBE-PBE functional in prediction of the B-N bond length in pristine BNNT, however, the long-range London dispersion effects, especially for the case weakly bonded vdW complexes were has a crucial effect on the interaction energies and thermophysical properties. There are several theoretical, empirical, and semiempirical methods to correct the dispersion effects, and previous studies proved that dispersion-corrected DFT methods can improve the accuracy of calculation of the van der Waals minima with the accuracy of about ± 0.2 kcal/mol^48^. We used the third generation of the Grimme’s empirical correction method in which the short-range damping effects has also been corrected using the Becke-Johnson damping function^39^ in combination with B3LYP^49–52^ functional to calculate the *E*_*ads*_ of the studied systems optimized at PBE-PBE/63-11G* level of theory. The calculated Eads values at B3LY-D3(BJ)/6-311G* level of theory have also been reported in Table 3.

**Table 3:** Adsorption Energies (E_ads_) for the studied complexes at PBE-PBE/6-311G* and B3LYP-D3 (BJ)/6-311G* levels of theory.

System	Cite	*E*_*ads*_ (PBE-PBE)	*E*_*ads*_ (B3LYP-D3(BJ))	DEV% ^a^
ADA/BNNT(5,5)	T1	− 14.07	− 18.40	23
	T2	− 6.75	− 18.70	64
	T3	− 7.09	− 10.13	30
	T4	− 15.71	− 19.60	20
ADA/BNNT(6,6)		− 8.00	− 11.75	47
ADA/BNNT(7,7)		− 6.47	− 10.44	61
ADA/BNNT(Al)		− 12.33	− 8.38	47
ADA/BNNT(Ga)		− 51.69	− 59.49	13
ADA/BNNT(P)		− 7.10	− 4.53	57
ADA/BNNT(As)		− 15.5	− 20.61	27

^a^DEV% = $DEV\% = 100|E_{ads} \left( {PBE - PBE} \right) - E_{ads} \left( {B3LYP - D3\left( {BJ} \right)} \right)/E_{ads} \left( {PBE - PBE} \right)|$, and AAD% = DEV% /n, where *n* = 8 is the number of considered complexes.

*E*_*ads*_ are in kcal/mol.

As can be seen, all of the calculated *E*_*ads*_ values are negative using both methods, which shows the adsorption process is exothermic, and the resulted ADA/nanotubes are more stable than the individual participants. The values of the *E*_*ads*_ for all cases, other than ADA/BN(Ga) are also in the range of physical adsorption. However, it seems that ADA molecule chemically bonded to the BN(Ga) surface. It is seen from Table 3 that, by increasing the radius of the nanotube, the tendency of the BNNT to adsorb the ADA molecule was decreased, so that, *E*_*ads*_ is in the order of BNNT(5,5) > BNNT(6,6) > BNNT(7,7). The result of Table 3 also shows that, the doping of Al and P elements, which belong to period 3 elements, reduces the *E*_*ads*_, whereas, for period 4 elements (i.e. As, and specially Ga) the *E*_*ads*_ was increased compared to the pristine BNNT. The overall feature of both methods is qualitatively in agreement, and shows a similar trend; however, in all cases, other than ADA/BN(P) complex, the *E*_*ads*_ values, calculated by B3LYP-D3(BJ) functional, are more negative than PBE-PBE results. The maximum of DEV% = 63.9% between these two methods was observed for T2 adsorption cite of pristine BNNT, and the minimum DEV% = 13% belongs to the ADA/BN(P) complex, for which, unlike all other cases, the *E*_*ads*_(PBE-PBE) is more negative than *E*_*ads*_(B3LYP-D3(BJ)). The average of the absolute deviation between these two methods was 5.04 kcal/mol, which is high enough for the case of weak vdW, and the absolute average deviation (AAD%) between two methods is 36%. In other words, inclusion of the London dispersion effects results is the more negative adsorption energies of the vdW complexes studied in this work, and therefore, all other calculations were done at B3LYP-D3(BJ)/6-311G* level of theory.

### Energetics properties

Some energetic parameters for the studied individual nanotubes and their complexes with ADA molecule at different positions were reported in Table 4. Different values of HLG were reported in the literature for armchair BNNT(5,5), which are depends on the size of the nanotube and the applied DFT methods. As examples, the value of 5.81 eV for B_75_N_75_H_20_ nanotube using HSE1PBE/6-31G method were reported by Rodríguez Juárez et al.^47^. Where, the value of 4.492 eV were reported by Doust Mohammadi and Abdullah^53^ for B_20_N_20_ armchair BNNT(5,5) at PBE0/6-311G(d) level of theory. Also, in our previous work^38^, we found that, the HLG and the other electronic properties are depends on the apllied theoretical methods, so that, for BNNT(5,5), BNNT(Al), and BNNT(Ga) the values of 6.28, 4.62, and 3.94 eV using B3LYP/6–311 + G* were obtained, respectively, and the values of 4.52, 3.14, and 2.51 eV were obtained at PBE-PBE/6–311 + G* level of theory. The values reported in Table 4 for BNNT(5,5) are for B_160_N_160_H_20_ structure at B3LYP-D3(BJ)/6-311G* level of theory, and are in agreement with our previous work^38^.

**Table 4:** The energies of HOMO (${\varepsilon }_{H}$) and LUMO (${\varepsilon }_{L}$), bond gap between HOMO and LUMO (HLG), chemical potential ($\mu$), chemical hardness ($\eta$), and electrophilicity ($\omega$).

System	Cite	ε _H_	ε _L_	HLG	*μ*	*η*	*ω*
BNNT(5,5)	BNNT	− 6.533	− 0.2834	6.2494	− 3.4082	3.1247	3.7173
	T1	− 6.151	0.0259	6.1771	− 3.0626	3.0885	3.0369
	T2	− 6.152	0.0263	6.1781	− 3.0627	3.0890	3.0365
	T3	− 6.021	− 0.3611	5.6594	− 3.1908	2.8297	3.5979
	T4	− 6.201	− 1.2572	4.9440	− 3.7292	2.4720	5.625
BNNT(6,6)	Cell	− 6.556	− 0.3096	6.2468	− 3.4330	3.1234	3.7734
	Complex	− 6.312	− 0.1238	6.1882	− 3.2179	3.0941	3.3466
BNNT(7,7)	Cell	− 6.550	− 0.3236	6.2265	− 3.4638	3.1132	3.7940
	Complex	− 6.331	− 0.1684	6.1628	− 3.2498	3.0814	3.4273
BNNT(Al)	Cell	− 6.481	− 1.4622	5.0184	− 3.9714	2.5092	6.2856
	Complex	− 6.297	− 1.2264	5.0710	− 3.7619	2.5355	5.5815
BNNT(Ga)	Cell	− 6.482	− 1.9334	4.5487	− 4.2077	2.2743	7.7847
	Complex	− 5.989	0.1261	6.1146	− 2.9312	3.0573	2.8103
BNNT(P)	Cell	− 6.57	− 0.9624	5.6070	− 3.7660	2.8035	5.0588
	Complex	− 5.867	− 1.0170	4.8501	− 3.4421	2.4250	4.8856
BNNT(As)	Cell	− 6.520	− 0.9872	5.5331	− 3.7537	2.7665	5.0932
	Complex	− 5.843	− 1.0893	4.7533	− 3.4660	2.3766	5.0546

All values are in (eV), and were obtained from completed nanotube and using B3LYP-D3(BJ)/6-311G* method.

It can be seen from Table 4 that, the HLG values for BNNT(5,5)/ADA complex at all T1-T4 positions was decreased relative to the individual BN nanotube. Also, an increase in the HLG reduction was observed for T4 position, for which the maximum of *E*_*ads*_ in ADA/BNNT complexes was observed. It is also observed that, for T1 and T3 positions the ${\varepsilon }_{H}$ was increased, where, for T2 and specially for T4 positions the ${\varepsilon }_{H}$ was decreased. High ${\varepsilon }_{H}$ reduction in T4 position reduced the band gap further, resulting in an increase in the metallic character, chemical potential and chemical hardness, and inversely, an increase in the electrophilicity of complex compared to the individual BNNT(5,5). HLG values of the pristine BNNT(5,5), BNNT(6,6), and BNNT(7,7) are also almost equal, which shows that, the change of the radius of the nanotube has not any considerable effect on the electronic properties of the BNNT. For BNNT(6,6)/ADA, and BNNT(7,7)/ADA complexes also a reduction in the HLG after adsorption of ADA was observed.

In the case of metal doped nanotubes (i.e., BN(Al) and BN(Ga)) an increase in the HLG after the adsorption of ADA molecules was observed, and the maximum of HLG was observed for ADA/BN(Ga) complex. The observed HLG increase, results in the reduction of metallic character, µ, and ω, and an increase in the chemical hardness. While, in the case of non-metallic doped BN(P) and B(As), this behavior is quite opposite. Where, in these cases, the HLG decreased, and therefore, chemical potential increased. Also both chemical hardness and electrophilicity were decreased.

**Figure 3:**
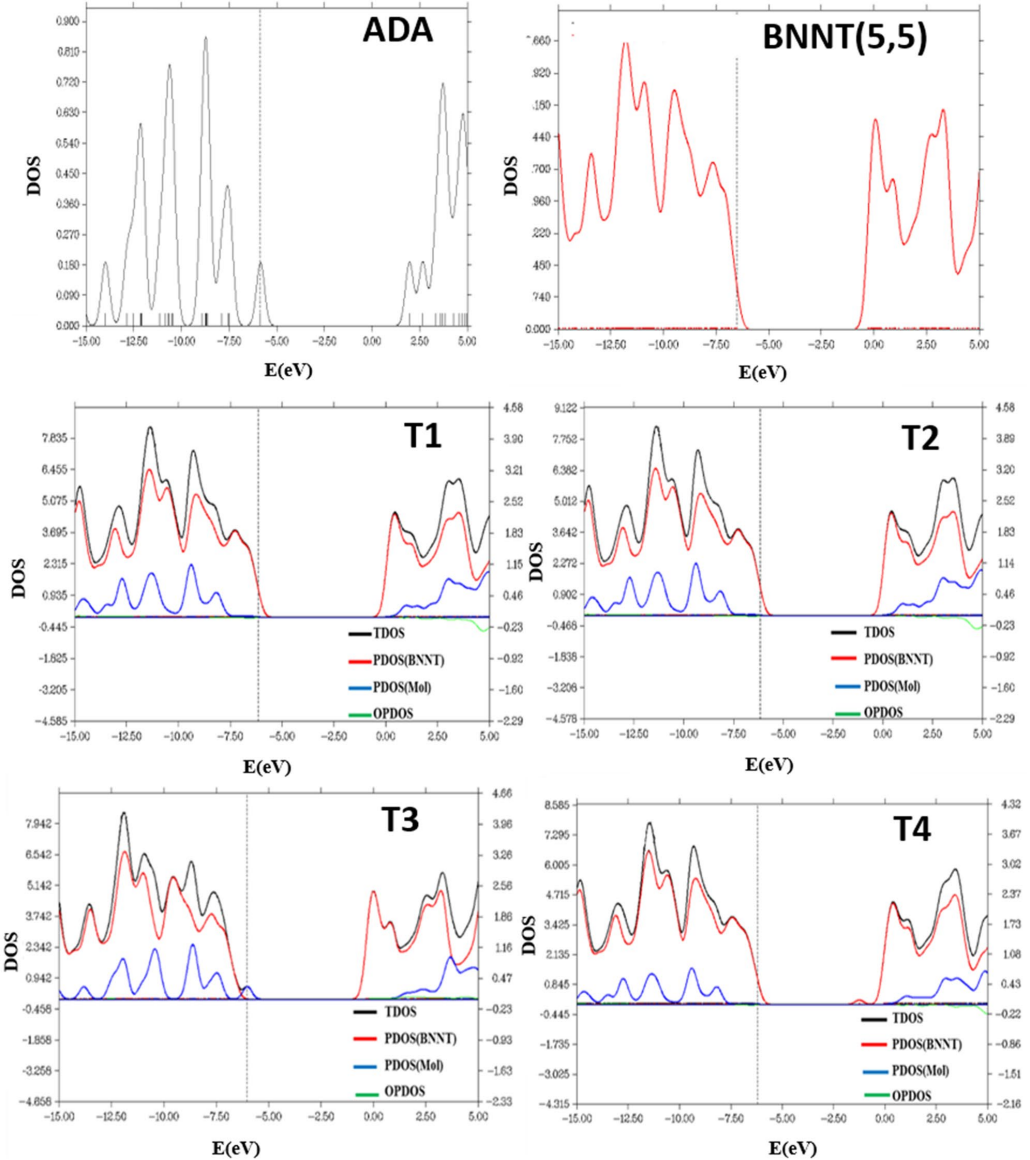
The density of state (DOS) spectra for the adsorption of ADA molecule onto the surface of the armchair BNNT(5,5).

**Figure 4:**
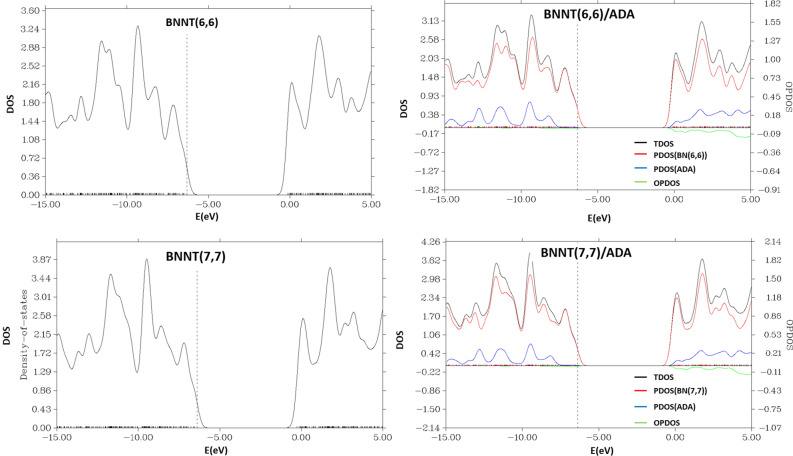
The density of state (DOS) spectra for the adsorption of ADA molecule onto the surface of the armchair BNNT(6,6), and BNNT(7,7).

**Figure 5:**
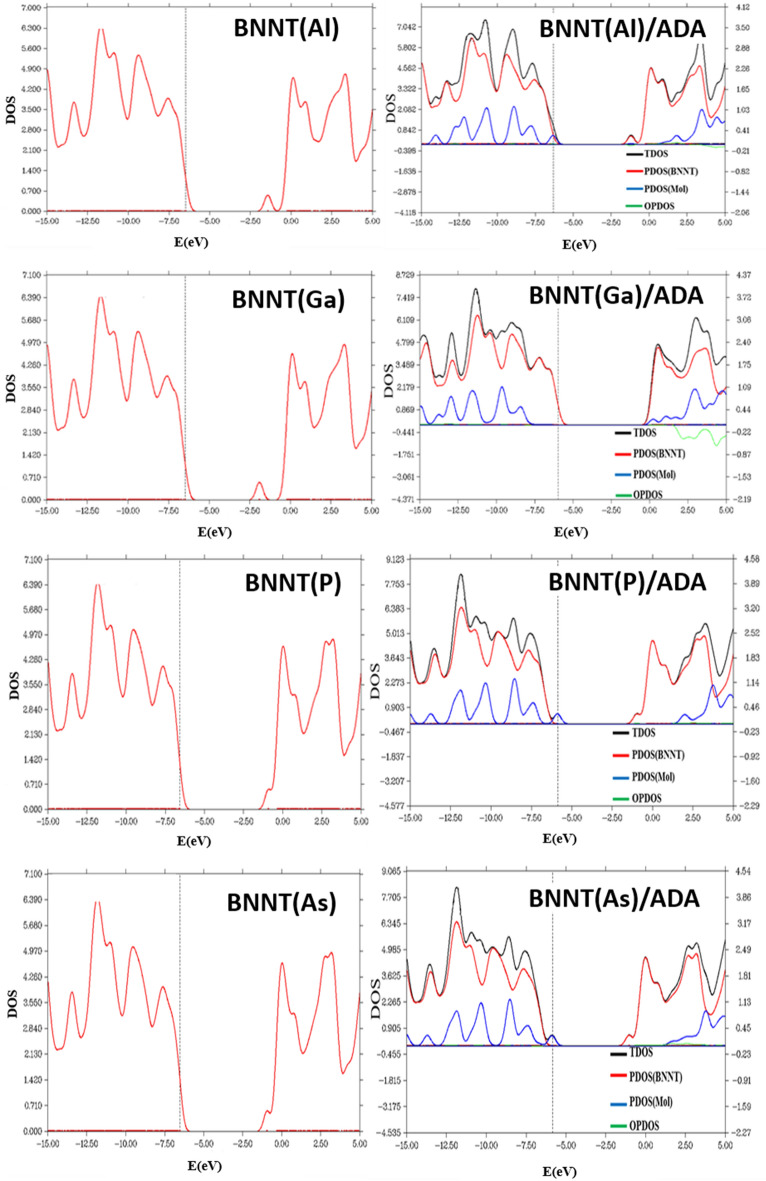
The density of state (DOS) spectra for the adsorption of ADA molecule onto the surface of the Aluminum (**a**), Gallium (**b**), Phosphorous (**c**), and Arsenic (**d**) doped boron nitride nanotube.

Density of states (DOS) of ADA molecule, each nanotube and ADA/ nanotube complexes along with the projected density of states (PDOS) for ADA and each nanotube were shown in Figs. 3, 4, 5. PDOS spectra shows the contribution of each individual ADA or nanotube in total DOS spectra of the complex. There is no distinguishable peak in the HLG region of T1 and T2 cases. In T1 and T2 cases the HOMO peak of ADA molecules was disappeared, and for T4 position a new peak in LUMO region was observed. However, there is not any significant change in T3 complex compared to the DOS of individual ADA on BNNT, which also in agreement with the lowest *E*_*ads*_ observed for this complex. Maximum of *E*_*ads*_ for ADA/BNNT(5,5) was observed for T4 complex, for which the most significant changes in DOS spectra, compared to its individual components, was observed. As shown in Fig. 4, the DOS spectra of the BNNT(6,6)/ADA, and BNNT(7,7)/ADA complexes are also similar to the T1–T3 adsorption positions of BNNT(5,5). The DOS spectra for the dopped cases were shown in Fig. 5. This figure shows that there is not a significant difference between DOS spectra of ADA/ nanotube complexes with individual components in BN(Al), BN(P) and BN(As) cases, which is also again confirms the physical adsorption of ADA on these nanotubes.

**Figure 6:**
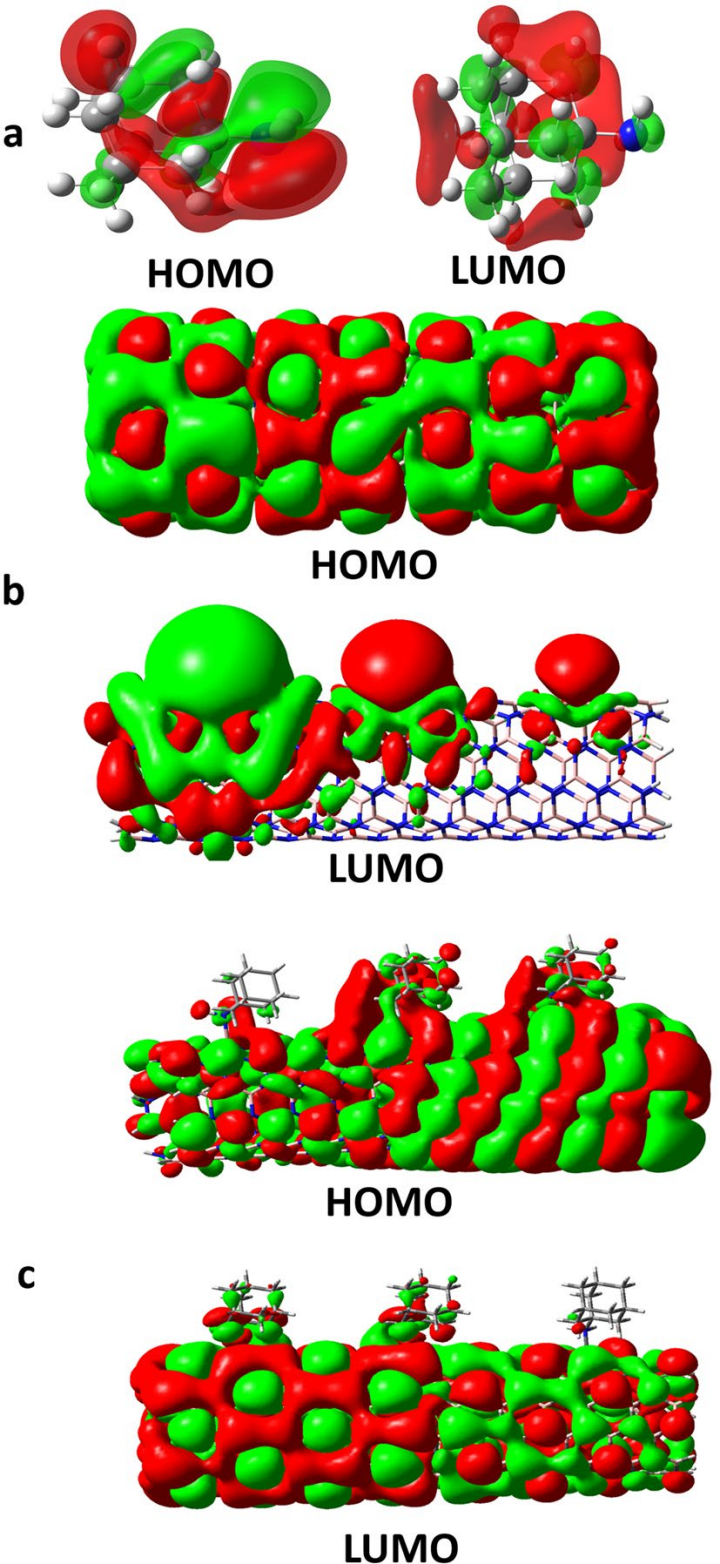
HOMO and LUMO energy level Kohn–Sham orbitals of (**a**) ADA molecule, (**b**) Ga-doped boron nitride nanotube, and (**c**) ADA/BNNT(Ga) complex.

In the case of ADA/BN(Ga) complex, however, the HOMO states related to ADA molecules and LUMO peaks belongs to BN(Ga) nanotubes were disappeared, and HLG was increased. Therefore, in this case, the electronic structure of the complex deserves effective changes, and *E*_*int*_ = –59.5 kcal/mol confirms the chemisorption of ADA on BN(Ga) nanotubes. The Kohn–Sham HOMO and LUMO molecular orbitals of ADA, BNNT(Ga) and their complexes have shown in Fig. 6. As can be seen, the HOMO orbital of BNNT(Ga) is mainly centered on Ga atom, however after the adsorption of ADA, the HOMO molecular orbital of ADA was mixed with LUMO state of BNNT(Ga), and made a chemical-bonding between ADA and BN(Ga). This analysis also confirms the results of DOS spectra.

### Bond length and bond orders

The Wiberg bond index (WBI)^54^ and bond length for possible bonds between ADA and BNNTs, and B-N bonds of BNNTs, as shown in Figs. 1 and 2, were reported in Table 5. From this table it is seen that, B-N bond length of BNNT(5,5), BNNT(6,6), and BNNT(7,7) were increased after the adsorption of ADA molecule, and also bond orders were decreased. Furthermore, the most important interaction in T1, T2 and T4 complexes of BNNT(5,5), along with the BNNT(6,6), and BNNT(7,7) is the interaction between the N of amino group of ADA and B atom of the BNNT. Whereas, for T3 complex the physisorption of ADA on BNNT is due to the hydrogen-bonding between H atoms of NH_2_ from ADA with N atom of BNNT. Again, for T1 and T2 positions almost similar results were obtained, (i.e., r_B-N_ = 1.78A^֯^ and WBI_B-N_ = 0.486), where, slightly stronger interaction of the same type was observed for T4 position, which has the maximum *E*_*ads*_.

**Table 5:** Bond length and Wiberg bond index (WBI) for pristine nanotubes before and after the adsorption of Amantadine.

System	Cite	State	Bond type	WBI^a^	Length
		Pristine NT	B–N	0.892	1.45
BNNT(5,5)	T1	NT	B–N1	0.729	1.53
			B–N2	0.722	1.52
			B–N3	0.744	1.51
		NT-AM	B–N(mol)	0.486	1.78
			N3–H1(mol)	0.005	2.43
	T2	NT	B–N1	0.729	1.53
			B–N2	0.722	1.52
			B–N3	0.744	1.51
		NT-AM	B3–N(mol)	0.486	1.78
			N2–H(mol)	0.005	2.43
	T3	NT	B–N1(NT)	0.863	1.45
			B–N2(NT)	0.859	1.46
			B–N3(NT)	0.861	1.46
		NT-AM	B1–H1(mol)	0.004	2.64
			N1–H2(mol)	0.001	2.96
	T4	NT	B–N1	0.736	1.54
			B–N2	0.737	1.52
			B–N3	0.709	1.51
		NT-AM	B–N(mol)	0.505	1.75
			N2–H2(mol)	0.002	2.62
BNNT(6,6)		Pristine NT	B–N	0.879	1.46
		NT	B–N1(NT)	0.716	1.52
			B–N2(NT)	0.721	1.52
			B–N3(NT)	0.737	1.51
		NT-AM	B3–N(mol)	0.453	1.81
			N1–H2(mol)	0.002	2.44
BNNT(7,7)		Pristine NT	B–N	0.880	1.46
		NT	B–N1(NT)	0.724	1.52
			B–N2(NT)	0.720	1.52
			B–N3(NT)	0.740	1.51
		NT-AM	B3–N(mol)	0.439	1.83
			N1–H2(mol)	0.002	2.45

Bond lengths and Bond orders for doped nanotubes, as shown in Fig. 1, and their complexes with ADA molecules were reported in Table 6. It is seen from this table that, the bond length of B-Al, B-Ga, P-B and As-B in doped nanotubes is greater than B-N bond of pristine BNNT(5,5), which is due to the superior atomic radius of doped atoms compared to the Boron and Nitrogen atoms. The relevant bond orders of metal doped nanotubes were decreased, where, for non-metallic cases an increase in the bond order was obtained.

**Table 6:** Bond length and Wiberg bond index (WBI) for Al, Ga, P, and As-doped nanotubes before and after the adsorption of Amantadine.

System	State	Bond type	WBI	Length
	pristine BNNT	B–N	0.8918	1.45
BNNT(Al)	Nanotube	Al–N1	0.5083	1.8
		Al–N2	0.4784	1.76
		Al–N3	0.4786	1.76
	Complex	N1–H1(ADA)	0.004	2.78
BNNT(Ga)	Nanotube	Ga–N1	0.559	1.84
		Ga–N2	0.559	1.8
		Ga–N3	0.595	1.8
	Complex	Ga–N(ADA)	0.312	2.06
		H1(ADA)–N1(NT)	0.006	2.51
		H2(ADA)–N2(NT)	0.004	2.66
BNNT(P)	Nanotube	P–B1	1.0403	1.96
		P–B2	1.0971	1.89
		P–B3	1.0971	1.89
	Complex	P–H(ADA)	0.0093	2.88
BNNT(As)	Nanotube	As–B1	1.0296	2.01
		As–B2	1.0865	1.96
		As–B3	1.0864	1.96
	Complex	As–H1(ADA)	0.0103	2.84

The only interaction between BN(Al) and ADA molecule is a weak hydrogen bonding between H of NH_2_ from ADA with N of BN(A) with r = 2.78 Å and WBI of only 0.004. However, for ADA/BN(Ga) complex, there is a strong interaction of chemical bonding order (WBI = 0.312) between N of NH_2_ and Ga, which strongly confirms the chemisorption of ADA on BN(Ga). In the case of ADA/BN(P) and ADA/BN(As) there is a weak hydrogen-bonding type of interaction between NH_2_ group of ADA molecule and P or As of nanotubes, for which, the BN(As) is stronger than BN(P), and therefor more exothermic in physisorption process.

### QTAIM analysis

**Figure 7:**
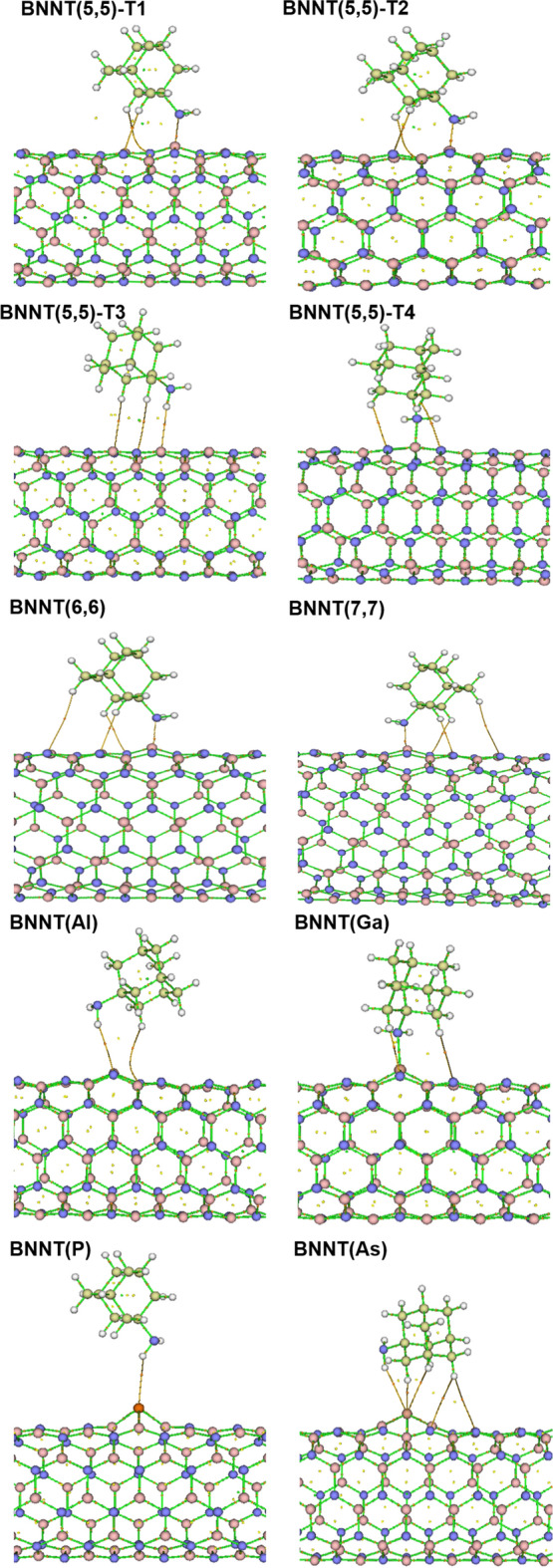
Bonds of ADA molecules with pristine and the doped nanotubes in QTAIM investigation.

The QTAIM analysis results have been reported in Table 7 and the critical points along with the possible bond paths were shown graphically in Fig. 7. In Table 7, ${\nabla }^{2}\rho (r)$ for all BCPS in T1-T4 complexes of BNNT(5,5)/ADA, along with the BNNT(6,6)/ADA, and BNNT(7,7)/ADA complexes, are positive, and ρ(r) ≤ 0.01. Therefore, the interaction is of non-covalent (vdW) type. For ADA/BN(Al) and ADA/BN(P) all positive interaction have ${\nabla }^{2}\rho \left(r\right)>0$, ρ(r) ≤ 0.008, and G(r)/V(r) > 1, which confirms the weak vdW interactions in these two cases. Two strong covalent bonds also observed for ADA/BN(As) (i.e., H4-N and H3-As) for which ${\nabla }^{2}\rho \left(r\right)<0$, ρ(r) > 0.27, G(r)/V(r) > 1, and the ellipticity of electrons are near zero. Other two interactions have negligible electron density, and are so weak that can be neglected. The most important interaction between ADA and BN(Ga) is the interaction between N atom of amino group of ADA, and Ga atom from nanotube. In this case ${\nabla }^{2}\rho \left(r\right)=0.293$ is the most positive Laplacian of $\rho \left(r\right)$ reported in this Table, also G(r)/|V(r)|= 0.83 and near zero ε values confirms that this interaction is of stable ionic-covalent mixed type.

**Table 7:** The QTAIM topological parameters, including electron density (ρ(r)), Laplacian of electron density (∇^2^ρ(r)), the kinetic electron density G(r), potential electron density V(r), eigenvalues of Hessian matrix (λ), and bond ellipticity index (ε) at BCPs of the ADA/nanotube complex systems.

system		Bond	ρ(r)	$\nabla^{2}$ ρ(r)	G(r)	V(r)	G/|V|	λ_1_	λ_2_	λ_3_	ε
**BNNT(5,5)**	T1	N–B(NT)	0.088	0.100	0.089	− 0.154	0.582	− 0.099	− 0.087	0.286	0.143
		H1–N(NT)	0.017	0.034	0.008	− 0.007	1.110	− 0.010	− 0.010	0.054	0.114
		H2–N(NT)	0.009	0.031	0.006	− 0.005	1.258	− 0.008	− 0.003	0.042	1.826
	T2	H1–N(NT)	0.010	0.030	0.006	− 0.005	1.179	− 0.008	− 0.007	0.044	0.139
		H2–N(NT)	0.008	0.027	0.006	− 0.005	1.242	− 0.004	− 0.002	0.033	0.983
		N–B(NT)	0.088	0.100	0.089	− 0.153	0.581	− 0.099	− 0.087	0.286	0.143
	T3	H1–N(NT)	0.008	0.024	0.005	− 0.004	1.174	− 0.007	− 0.007	0.037	0.103
		H2–N(NT)	0.005	0.014	0.003	− 0.002	1.347	− 0.004	− 0.003	0.021	0.403
		H3–B(NT)	0.004	0.011	0.002	− 0.002	1.361	− 0.003	− 0.003	0.017	0.125
	T4	N–B(NT)	0.096	0.144	0.106	− 0.176	0.602	− 0.119	− 0.109	0.371	0.093
		H1–N(NT)	0.010	0.030	0.006	− 0.005	1.179	− 0.008	− 0.007	0.044	0.139
		H2–N(NT)	0.008	0.027	0.006	− 0.005	1.242	− 0.004	− 0.002	0.033	0.983
**BNNT(6,6)**		N–B(NT)	0.084	0.076	0.075	− 0.132	0.572	0.231	− 0.073	− 0.083	0.140
		H1–N(NT)	0.011	0.033	0.007	− 0.006	1.212	0.050	− 0.008	− 0.010	0.215
		H2–N(NT)	0.009	0.028	0.006	− 0.005	1.208	0.038	− 0.003	− 0.007	1.355
**BNNT(7,7)**		N–B(NT)	0.080	0.059	0.068	− 0.121	0.561	0.199	− 0.075	− 0.065	0.157
		H1–N(NT)	0.010	0.031	0.007	− 0.005	1.215	0.047	− 0.009	− 0.007	0.265
		H2–N(NT)	0.001	0.003	0.001	0.000	1.849	0.004	0.000	0.000	0.198
**BNNT(Al)**		H1–N(NT)	0.005	0.011	0.002	− 0.002	1.237	− 0.003	− 0.002	0.016	1.074
		H2–N(NT)	0.007	0.021	0.004	− 0.003	1.288	− 0.006	− 0.003	0.030	0.753
**BNNT(Ga)**		H1–N(NT)	0.009	0.025	0.005	− 0.005	1.160	− 0.007	− 0.007	0.039	0.071
		H2–N(NT)	0.012	0.034	0.008	− 0.007	1.098	− 0.010	− 0.008	0.054	0.285
		N–Ga	0.078	0.293	0.091	− 0.110	0.832	− 0.094	− 0.093	0.480	0.005
**BNNT(P)**		H–P(NT)	0.008	0.025	0.005	− 0.004	1.275	− 0.007	− 0.007	0.039	0.023
**BNNT(As)**		H4–N(NT)	0.271	0.874	0.166	− 0.114	− 1.456	− 0.001	− 0.001	0.012	0.075
		H4–B(NT)	0.311	0.105	0.194	− 0.124	− 1.565	− 0.002	− 0.001	0.013	0.875
		H3–As	0.749	0.206	0.409	− 0.303	− 1.350	− 0.005	− 0.005	0.030	0.013
		H2–As	0.121	0.351	0.765	− 0.652	− 1.173	− 0.009	− 0.008	0.053	0.078
		H1–As	0.912	0.262	0.538	− 0.42	− 1.281	− 0.007	− 0.007	0.040	0.033

All values have been obtained at B3LYP-D3(BJ)/6-311G* level of theory.

### NBO results

The electron donor–acceptor configurations on the basis of natural bond orbitals (NBO) along with their second perturbative stabilization energies (E^2^) for pristine and doped nanotubes, and all of the studied ADA/nanotube complexes were reported in Tables 8 and 9, respectively. From Table 8, it can be inferred that, for all doped nanotubes, the most stable interactions are between the doped atom and the nanotube. The existence of such interaction with large E^2^ values confirms that the added Al, Ga, P, and As atoms is really doped to the structure of the nanotube with strong chemical bonds. The data reported in Table 9 also shows that, for ADA/BNNT(5,5), ADA/BNNT(6,6), and ADA/BNNT(7,7) cases, the most important donor–acceptor interaction with the highest E^2^ value is the transfer of electron from bonding BD(B-N) state as donor to the antibonding BD*(B(NT)-N(ADA)) as acceptor. Also, the accumulate E^2^ values for T4 position is greater than the other three cases, which confirms the strongest adsorption in T4 position. For doped complexes, on the other hand, the strongest Lewis acid–base interaction belongs to the case of metal-doped nanotubes, and specially BN(Ga), in which the metal atom plays the role of Lewis acid, and the N atom of amino group of ADA shows the Lewis base characteristics.

**Table 8:** The doner-acceptor NBO interactions and second order perturbation estimation of the stabilization energy (E^2^) for the boron nitride nanotubes, calculated at B3LYP-D3(BJ)/6-311G* level of theory.

System	Donor	Acceptor	E^2^(kcal/mol)
BNNT(5,5)	LP(N)	BD*(N–B)	50.94
	BD(N–B)	LP*(B)	52.15
BNNT(6,6)	LP(N)	BD*(N–B)	50.1
	BD(N–B)	LP*(B)	47.93
BNNT(7,7)	LP(N)	BD*(N–B)	49.02
	BD(N–B)	LP*(B)	46.99
BNNT(Al)	LP(N1)	LP*(Al)	88.51
	LP(N2)	LP*(Al)	88.29
	LP(N3)	LP*(Al)	83.43
	LP(Al)	BD(B–N)	4.6
BNNT(Ga)	BD(N–Ga)	BD*(N–B)	43.99
	BD(N–Ga)	BD*(N–B)	39.69
	BD(N–B)	LP*(Ga)	17.15
	BD(N–B)	BD*(N–Ga)	14.24
	BD(N–B)	BD*(N–Ga)	4.01
	BD(N–B)	BD*(N–Ga)	2.86
BNNT(P)	BD(N–B)	BD*(B–P)	3.39
	BD(N–B)	BD*(B–P)	3.03
	BD(N–B)	BD*(B–P)	2.86
	BD(N–B)	BD*(B–P)	1.84
BNNT(As)	LP(As)	BD*(B–N)	8
	BD(B–As)	BD*(B–N)	4.93
	BD(B–As)	BD*(B–N)	3.2
	BD(B–N)	BD*(B–As)	1.74

**Table 9:** The donor–acceptor NBO interaction and second order perturbation energy (E^2^) for the ADA/nanotube complexes, calculated at B3LYP-D3(BJ)/6-311G* level of theory.

System	Cite	Donor	Acceptor	E^2^(kcal/mol)
BNNT(5,5)	T1	BD(B–N)	BD*(B–N(mol))	14.35
	T2	BD (B–N)	BD*(N–H)	1.3
	T3	BD(N–B)	BD*(B–N(mol))	14.36
		BD(B–N)	BD*(B–N(mol))	11.74
	T4	BD(B–N)	BD*(B–N(mol))	12.86
		BD(B–N)	BD*(B–N(mol))	12.51
		BD(N–B)	BD*(B–N(mol))	11.83
	BNNT(6,6)	BD(N–H)	LP*(B)	17.61
		BD(N–H)	LP*(B)	16.90
		BD(C–N)	LP*(B)	11.74
	BNNT(7,7)	BD(N–H)	LP*(B)	16.53
		BD(N–H)	LP*(B)	15.83
		BD(C–N)	LP*(B)	10.85
	BNNT(Al)	LP(N^a^)	LP*(Al)	85.46
		LP(N^a^)	LP*(Al)	81.7
		LP(N^a^)	LP*(Al)	80.77
	BNNT (Ga)	LP(N^a^)	LP*(Ga)	122.53
		LP(N^a^)	LP*(Ga)	121.08
		LP(N^a^)	LP*(Ga)	118.61
		LP(N^a^)	LP*(Ga)	72
	BNNT (P)	LP(P)	BD*(B–N)	10.28
		LP(P)	BD*(B–N)	10.06
		BD(B–P)	BD*(N–B)	4.79
		BD(B–P)	BD*(N–B)	3.68
		LP(P)	BD *(N–H)	3.06
	BNNT (As)	LP(As)	BD*(N–H)	1.97
		LP(As)	BD*(C–H)	1.04
		BD(C–H)	BD*(B–As)	0.95

## Conclusions

This work was devoted to study the interaction of ADA with pristine BNNT(5,5), BNNT(6,6) and BNNT(7,7) along with the Al, Ga, As, and P doped BN nanotubes, using density functional theory methods. The effect of long-range dispersion effects was studied using B3LYP-D3(BJ) empirical method, and it was found that, the elimination of this effect may cause an error of about 65% in absolute deviation of the calculated bonding energies, and therefore in the case of van der Waals (vdW) complexes with weak physical interactions should be considered. It was found that, the adsorption energy (*E*_*ads*_) for pristine BNNTs is in the order of BNNT(5,5) > BNNT(6,6) > BNNT(7,7), which shows that, *E*_*ads*_ was decreased by increasing the radius of the armchair BNNT. Where, the cohesion energy (*E*_*coh*_) is in the opposite direction, and increased by increasing the radius of BNNT. The results have also showed that, metallic Al and Ga, and non-metallic P and As can be doped in the structure of BNNT(5,5), without the destruction of the nanotube, and the *E*_*coh*_ of the all dopped nanotubes is about 0.04 eV/atom positive than the pristine BNNT(5,5). Also, ADA molecule physically adsorbed on pristine BNNT(5,5), BNNT(6,6), BNNT(7,7), BN(As), BN(P) and BN(Al) nanotubes, and the ADA/NT interactions are of weak vdW type in these cases. Therefore, these nanotubes are acceptable candidates to design ADA nano-sensors for drug delivery purposes. However, BN(Ga) nanotube chemically absorbs ADA via Lewis acid base interaction, and therefore, the low yield drug release, and the poisoning of the BN(Ga) based nano-sensor, made the use of BN(Ga) nanotube with serious problems toward ADA drug.

## Data Availability

All data generated or analyzed during this study are included in this published article.
